# Thyroid Function and Sleep Patterns: A Systematic Review

**DOI:** 10.7759/cureus.63447

**Published:** 2024-06-29

**Authors:** Sunaina Addanki, Krina Patel, Lisa Patel, Blake Smith, Prem Patel, Sadhika Uppalapati, Lubov Nathanson

**Affiliations:** 1 Medicine, Nova Southeastern University Dr. Kiran C. Patel College of Osteopathic Medicine, Fort Lauderdale, USA; 2 Osteopathic Medicine, Nova Southeastern University Dr. Kiran C. Patel College of Osteopathic Medicine, Fort Lauderdale, USA; 3 Medicine, Nova Southeastern University, Fort Lauderdale, USA; 4 Medicine, Nova Southeastern University Dr. Kiran C. Patel College of Allopathic Medicine, Fort Lauderdale, USA

**Keywords:** autoimmune hypothyroidism, thyroid function, sleep pattern, chronic insomnia, clinical hypothyroidism

## Abstract

Hypothyroidism, defined as a low metabolic function of the thyroid gland that results in low thyroid hormone levels, and insomnia, a condition with the inability to sleep, are two distinct conditions with little overlap that have been extensively established. Both conditions have been studied independently in terms of epidemiology, pathophysiology, diagnosis, and management. The exact causal relationship between the two conditions has yet to be elucidated, and a direct underlying pathophysiology has not been pinpointed. To gain further insight into the relationship between hypothyroidism and insomnia, we performed a systematic review to explore this relationship using predetermined guidelines. Out of 59 studies assessed, four studies evaluated the mechanisms of these two potentially comorbid conditions. Our findings suggest that hypothyroidism and insomnia may have a bidirectional relationship, with symptomatic overlap that is tied to increased metabolic comorbidities and hormonal dysregulation. These findings warrant further research to verify these early findings and gain further insight into the relationship between these conditions. A better understanding of the pathophysiology of overlap between these two conditions will help improve diagnosis and target treatment more effectively.

## Introduction and background

Hypothyroidism is characterized by an underactive thyroid gland, resulting in the insufficient production and release of thyroid hormones. The primary hormones, thyroxine and triiodothyronine, are secreted from the thyroid gland, which plays a crucial role in the regulation of the body’s metabolism. Although the prevalence of hypothyroidism can vary by age, sex, and geographical location, approximately 5% of the overall population is impacted by hypothyroidism, and an additional 5% are believed to be undiagnosed [1]. Hypothyroidism generally presents with fatigue, weakness, and weight gain [2]. Other common symptoms include dry skin/hair, muscle aches, difficulty tolerating colds, and difficulty sleeping. While the exact incidence of hypothyroidism varies depending on the population studied and the diagnostic criteria used, approximately one in 20 Americans, ages 12 and older, have hypothyroidism. Generally diagnosed by blood tests that measure one’s levels of thyroid-stimulating hormone (TSH) and thyroxine (T4), hypothyroidism is treated using hormone replacements such as levothyroxine [3].

More recent studies have identified a link between hypothyroidism and sleeping disorders, with possible proposed mechanisms being elucidated [4]. Insomnia is a common sleep disorder characterized by difficulty falling and/or staying asleep. Patients with insomnia may suffer from this condition despite having sufficient opportunity for sleep. This condition is linked to severe distress or impairment in daytime functioning [5]. The prevalence of insomnia has been shown to range from 10 to 30% in the general population [6]. Although insomnia affects individuals of all ages, it is highly prevalent in older women going through peri- and post-menopause [7]. Approximately 31 to 42% of peri-menopausal women experience insomnia [8]. In many cases of insomnia, co-occurring physical conditions, such as arthritis, fibromyalgia, headaches, and orofacial pain, have been identified [9]. Symptoms of insomnia typically include complaints of fatigue, daytime sleepiness, mood swings, exhaustion, cognitive impairment, and impaired occupational and social functioning [10]. Individuals who suffer from chronic insomnia face disrupted sleep more than three times a week for three months or longer [5]. An inability to fall asleep, stay asleep, or wake up early, along with the resulting dysfunction during the day, is the basis for a clinical diagnosis of insomnia [11]. Both non-pharmacologic therapies, such as cognitive behavioral therapy, and pharmacologic therapies, like “Z-drugs,” benzodiazepines, melatonin receptor agonists, orexin antagonists, antipsychotics, and antidepressants, are available to treat insomnia [5].

While a definitive biological link between insomnia and hypothyroidism has yet to be established, there are several proposed mechanisms linking the two conditions. There are several shared characteristics between the two conditions, which may be due to the bidirectional relationship these conditions may have with one another. Common symptoms of hypothyroidism, such as prolonged sleep latency, shorter duration of sleep, and lower overall sleep satisfaction, are all characteristics that may be contributing to insomnia [12]. Additionally, individuals with hypothyroidism who experience symptoms, such as myalgias, joint pain, or anxious mental states, may be due to insomnia rather than solely hypothyroidism [12]. This strong overlap in symptoms has yet to be explored in detail.

TSH is a hormone derived from the hypothalamus-pituitary-thyroid axis and is known to be influenced by the circadian rhythm. The circadian rhythm of TSH and thyroid hormone secretion is influenced by both the quality and duration of sleep. Conversely, TSH and thyroid hormones play a significant role in affecting sleep quality and parameters [4]. Abnormal TSH levels found in hypothyroid patients, along with TSH’s connection to the circadian rhythm, could also be a likely explanation for the prevalence of insomnia and other sleep disturbances among individuals with hypothyroidism. Low circulating levels of thyroid hormone may disrupt the proper functioning of the circadian rhythm, ultimately leading to sleep disturbances [12]. Another proposed mechanism to explain the association of hypothyroidism with insomnia is due to their clinical correlations with shared medical comorbidities [12]. The insomnia that hypothyroid individuals experience might be attributed to medical comorbidities that arise from an irregular thyroid gland rather than solely the abnormal hormone levels themselves. Medical comorbidities that may arise from an irregular thyroid gland, such as obstructive sleep apnea and restless leg syndrome, are implicated in the disruption of adequate sleep and have strong associations with both hypothyroidism and insomnia alike. Obstructive sleep apnea and restless leg syndrome may potentially be the root cause in implicating insomnia’s association with hypothyroidism [12]. In one study, Green and colleagues emphasized a whole-body approach to explain the relationship between hypothyroidism and insomnia.

Hypothyroidism and insomnia have been extensively studied as independent conditions in terms of epidemiology, pathophysiology, diagnosis, and management. Despite the bidirectional relationship between the two conditions remaining undetermined with no direct underlying pathophysiology being identified, some research has demonstrated that hypothyroidism can lead to poor sleep quality. The aim of this study was to explore the complex relationship between hypothyroidism and insomnia. A deeper comprehension of this complex relationship will help facilitate improved diagnosis and, more precisely, targeted treatment strategies. These findings may potentially hold promise in improving future care and quality of life for those with hypothyroidism.

## Review

Methods

Inclusion/Exclusion Criteria

The inclusion criteria for the systematic review were English-written articles published in peer-reviewed journals specifically assessing the association between hypothyroidism and insomnia. This manuscript excluded articles that did not directly address the relationship between hypothyroidism and insomnia. It also excluded articles that were unable to be converted to a full-text format, such as abstracts or summaries that lacked sufficient details. Articles that were not written in English or articles that were unable to be translated into English were excluded as well. Studies were also excluded if they were duplicates or had restricted access rights.

Information Sources

**Figure 1:**
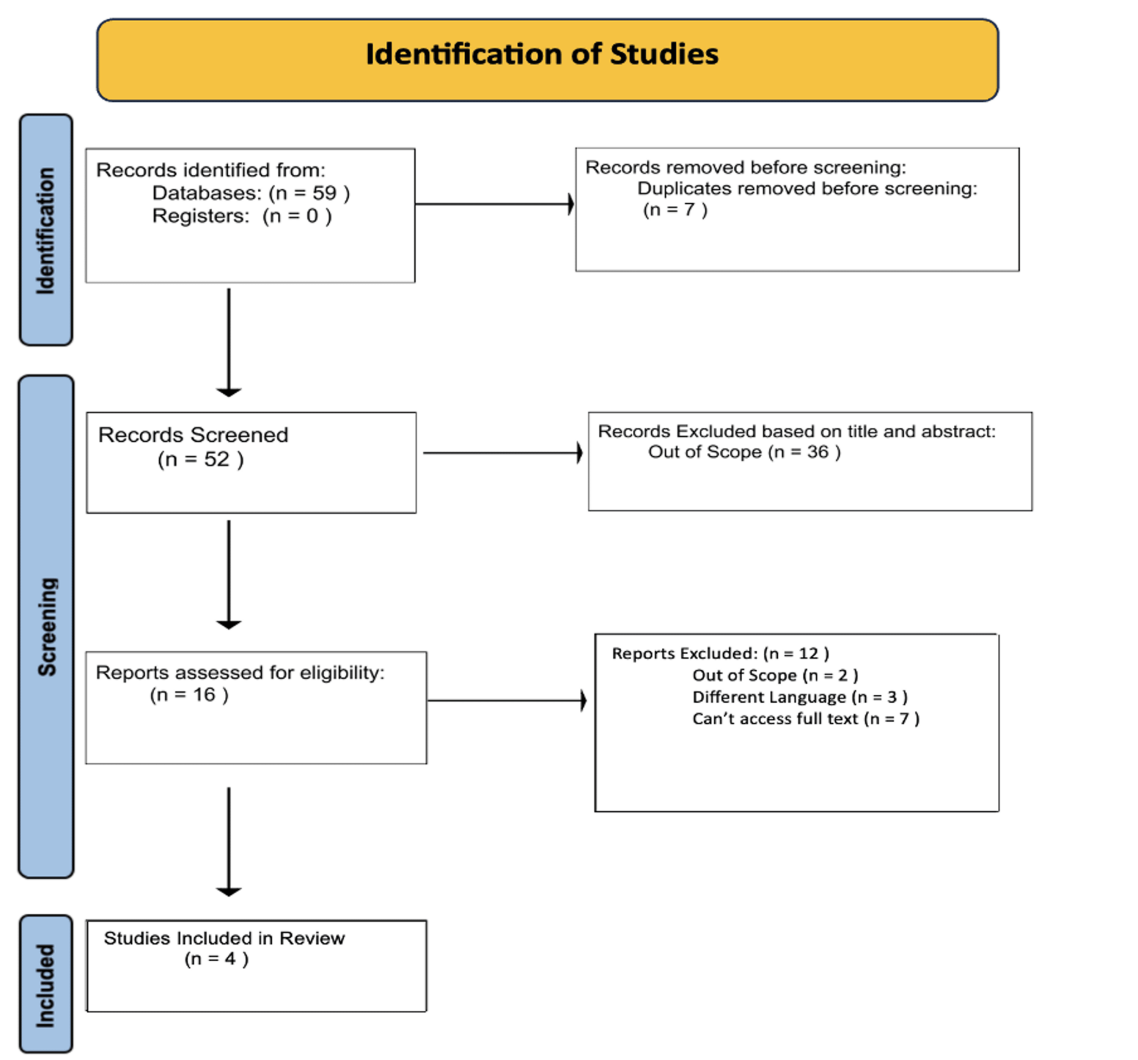
Article selection diagram

In order to find recent data on hypothyroidism and insomnia, a thorough literature review was conducted. A comprehensive search strategy targeting only published and difficult-to-find literature was employed to identify potential studies relevant to our review. The databases EBSCO host, Embase, PubMed, and Google Scholar were utilized to find the articles using key terms such as “hypothyroidism,” “insomnia,” “sleep disorders,” “sleep disturbance,” and “Hashimoto thyroiditis.” All the database queries were completed in October 2023 and were consistent throughout searches. The search consisted of 59 total studies for review. Three of the members performed an initial screening by viewing the titles and abstracts of the articles and deciding which articles were to be uploaded to Rayyan [13]. Figure 1 provides a step-by-step explanation of the selection process.

Search Strategy

Our primary objective was to compile scholarly articles and research findings that explored the potential associations, mechanisms, and implications of hypothyroidism on insomnia, and vice versa, as well as its underlying mechanisms of action. Data collection began on October 7, 2023, and was completed on October 18, 2023, with the databases searched being EBSCOhost, Embase, PubMed, and Google Scholar. A variety of databases were used to ensure that relevant literature from multiple fields and a broader range of journals and publication types were captured. The research team used several key terms, including “insomnia,” “hypothyroidism,” “sleep disorder,” “sleep disturbances,” and “Hashimoto thyroiditis” to ensure substantial relevance of the publications to the review. These terms were paired with Boolean operators, such as “and,” “or,” and “not,” to refine and broaden searches. For example, "hypothyroidism AND insomnia" or “hypothyroidism NOT hyperthyroidism” focus on studies that specifically address the relationship between the two conditions. Publications only written in the English language were included. To ensure consistency, all reviewers screened the same 52 articles after removing seven duplicates from a total of 57 articles using Rayyan. Any of the articles the reviewers disagreed upon or considered “maybe” were discussed and voted upon to avoid any discrepancies. If necessary, any disagreements that could not be resolved were referred to other reviewers. Thirty-six of those articles were removed based on the title or abstract being out of scope from our discussion. With 16 articles remaining, 12 articles were excluded due to language barriers, access barriers, and out-of-scope reports, leaving four articles. The articles were presented in a table highlighting the key findings and limitations of each article (Figure 1).

Results

Final screening of all articles resulted in four articles that evaluated the bidirectional relationship between hypothyroidism and insomnia. One article attributes this connection to low thyroid levels causing muscle/joint pain, cold intolerance, and anxiety, which may instigate increased sleep latency, difficulty sleeping, and excessive daytime sleepiness [12]. A cross-sectional study conducted among individuals of Indian descent found that daytime sleepiness and snoring had a significant correlation with impaired functioning of thyroid hormones [14]. Similarly, a retrospective study that evaluated female patients with hypothyroidism found that early chronotype, defined as those with a propensity for early awakenings, had higher TSH levels in the early evening and the middle of the night [15]. These higher levels of TSH secretion shift the circadian clock to a state of “morningness,” resulting in early awakenings [16]. In mild hypothyroidism, the TSH secretion pattern can be unaffected, but with significantly elevated levels of TSH, the circadian rhythm is rendered nonfunctional. Nocturnal sleep deprivation disinhibits the release of TSH, resulting in higher levels of morning TSH in those with insomnia and disrupted sleep patterns [17]. Findings from each of the four articles are included (Table 1). 

**Table 1:** Studies on thyroid dysfunction and sleep disorders TSH, thyroid stimulating hormone

Article	Summary (Findings)
Title: Thyroid Dysfunction and Sleep Disorders; Authors: Green, Max E; Bernet, Victor; Cheung, Joseph [12]; Study Type: Literature Review	High levels of thyroid hormone were associated with several components of sleep dysfunction, including prolonged sleep latency, difficulty maintaining sleep, and excessive daytime sleepiness. Low thyroid hormone levels/subclinical hypothyroidism lead to longer sleep latency, shorter sleep duration, and lower satisfaction with their sleep quality compared with euthyroid individuals. Limitations: Small sample sizes in previous studies were referenced.
Title: Primary Hypothyroidism and Chronotypes in Adult Women; Authors: Arosemena MA; Ramos AR; Marcus EN; Slota KA; Cheung J; Castillo PR [16]; Study Type: Retrospective Study	Evaluated chronotypes of 99 female patients with hypothyroidism and stratified into an early, intermediate, or late chronotype group. Significant differences were found between patients in the early and late/intermediate chronotype groups with respect to sleep duration (7.30 vs. 7.04), body mass index (29.4 vs. 31.1), and TSH level (2.89 vs. 1.69), respectively. Limitations: Small sample size limits implications to larger populations. The study included a number of obese patients who could possibly have undiagnosed obstructive sleep apnea.
Title: Interconnection Between Circadian Clocks and Thyroid Function; Authors: Ikegami K; Refetoff S; Van Cauter E; Yoshimura T [17]; Study Type: Literature Review	Evaluated the link between circadian clocks and thyroid function to highlight a possible link between disrupted cardiac machinery and thyroid cancer. Daily TSH secretion profiles are typically disrupted in individuals with hypothyroidism and hyperthyroidism. The central circadian pacemaker in the hypothalamic suprachiasmatic nucleus controls peripheral circadian clocks. Chronic circadian disruption, such as from shift work or irregular sleep patterns, is associated with long-term health consequences, including obesity, type-2 diabetes, and cancer. Limitations: Mechanisms generating TSH circadian rhythms are still unclear.
Title: Prevalence of Sleep Abnormalities and Their Association Among Hypothyroid Patients in an Indian Population; Authors: Krishnan PV; Vadivu AS; Alappatt A; Kameswaran M [14]; Study Type: Cross-sectional study	Assessed the occurrence of sleep irregularities and their connection to hypothyroidism/metabolic risk factors in a comparatively lean urban population of 26,000 individuals from South India. Male individuals, individuals of older age, smokers, and individuals with higher body mass index, neck circumference, blood pressure levels, and hypothyroidism were more likely to be snorers. Limitations: Self-reported data with generic questions limited the analysis and quality of sleep-related disorders.

Discussion

Multiple research papers analyzed the intricate interplay between thyroid function and sleep disorders overall. While a modest amount of literature exists exploring the relationship between hypothyroidism and insomnia, potential mechanisms contributing to the heightened risk of concurrent hypothyroidism and insomnia have been hypothesized. These mechanisms stem from symptomatic manifestations of hypothyroidism, elevated risks of metabolic comorbidities in hypothyroid patients, and hormonal dysregulation, inducing alterations in both circadian rhythms and respiratory centers.

In one study, patients with hypothyroidism indicated they had longer sleep latency periods, shorter sleep duration, and lower satisfaction with sleep quality. Proposed mechanisms for these findings include the wide range of thyroid dysfunction symptoms exacerbating sleeping difficulties, possibly inducing insomnia. For example, underactive thyroid function is associated with muscle and joint pain, cold intolerance, and increased anxiety, all of which have been shown to contribute to insomnia [12]. Another study indicated that patients with hypothyroidism are at increased risk for a higher number of medical comorbidities, which increases the risk of insomnia. This study focused on obese women of South Asian descent who were found to have significantly increased daytime sleepiness and impaired thyroid function. It has been found that hypothyroid patients and obese patients have depression of the respiratory center and altered bellows, affecting their thorax and sleeping patterns [14].

Additional studies also prompted broader considerations for hormonal regulation shaping sleeping patterns as correlations between TSH levels, circadian preferences, and early chronotypes were made. Higher TSH levels present in hypothyroidism were found to be correlated with early chronotypes, possibly shifting the circadian clocks toward “morningness.” This may be due to higher TSH secretions toward early evenings and nighttime. Ultimately, early chronotypes may lead to findings of insomnia [16]. The role of the suprachiasmatic nucleus was concurrently explored in the complex relationship between hypothyroidism and insomnia.

Another study investigated the relationship between the suprachiasmatic nucleus in patients with hypothyroidism and its impact on their sleeping patterns [18]. The suprachiasmatic nucleus within the hypothalamus drives the expression of vital homeostatic functions such as feeding, body temperature, and neurohormone secretions, playing a vital role in sleep-wake rhythms [19]. This study found that individuals with hypothyroidism had significant disruptions in the suprachiasmatic connectivity between the suprachiasmatic nucleus, the precuneus, and the hypothalamus. The precuneus structure has been identified to have a significant role in sleep disturbances, especially among Alzheimer’s patients [20]. The diminished connectivity between these regions was proposed to affect neurotransmitter and hormonal feedback loops essential for normal circadian function, underscoring the potential role of hormonal dysregulation in insomnia [19].

While limited studies investigated the role of hypothyroidism in insomnia, a faulty circadian clock was also found to increase the risk of thyroid disease in a tissue-specific manner, making this relationship bidirectional [17]. Diminished T4 levels in central hypothyroidism, which may be caused by TRH deficiency-induced abnormal glycosylation of TSH. This has been indicated in the loss of normal circadian pattern of TSH secretion, leading to sleeping abnormalities [17]. A more recent cross-sectional and longitudinal study investigated the association between thyroid function and sleep quality [21]. The proportions of poor sleep and occasional poor sleep in subjects with isolated TSH elevation were significantly higher than those with normal TSH levels (70.24% vs. 49.58%, p = 0.001; 9.52% vs. 1.68%, p = 0.006) [21]. This study also concluded that isolated elevated TSH concentrations normalize when sleep status is improved, indicating the bidirectional relationship that may be evident between hypothyroidism and insomnia [21].

As highlighted, there are several potential mechanisms that offer insight into the connection between hypothyroidism and insomnia, yet much remains to be unveiled and explored in this intricate relationship.

Limitations

This systematic review is associated with several limitations, which primarily stem from our inclusion and exclusion criteria, and multiple articles were excluded from our analysis. This could have led to the exclusion of valuable data and diverse population groups. In addition, a constraint of our study lies in the scarcity of research focusing specifically on the relationship between hypothyroidism and insomnia. Many studies instead focused on general sleeping variations or difficulties, deviating from the medical definition of insomnia. Given this continually developing understanding of the relationship between hypothyroidism and insomnia, future studies should emphasize identifying causation and correlation between these conditions. Consequently, delving deeper into the underlying biochemical and genetic mechanisms may provide a better understanding of the two conditions occurring concurrently. Through this, the identification of disease biomarkers may hold promise for developing targeted interventions to alleviate these issues.

## Conclusions

The reviewed research articles highlighted an intricate potential relationship between hypothyroidism and insomnia that may be influenced by a multitude of factors. The disruptions in sleep patterns among individuals with hypothyroidism indicate the significant impact of thyroid dysfunction and sleep function. This comprehensive review offers various mechanisms linking the two conditions bidirectionally, including symptomatic manifestations, metabolic comorbidities, and hormonal dysregulation. Acknowledging the limitations of this study, future studies should target deeper explorations of underlying biochemical and genetic mechanisms that may provide a better understanding of the pathophysiology of the two conditions occurring in concert. It may lead to the identification of biomarkers of disease activity that may help to target treatment more effectively. In addition, longitudinal studies tracking individuals with hypothyroidism and the progression of insomnia symptoms may provide temporal dynamics that may lead to more advanced interventions.
